# The Therapeutic Potential of Propranolol and Other Beta-Blockers in Hyperthyroidism

**DOI:** 10.3390/ijms26178322

**Published:** 2025-08-27

**Authors:** Weronika Szybiak-Skora, Miłosz Miedziaszczyk, Edyta Szałek, Katarzyna Lacka

**Affiliations:** 1 University Clinical Hospital in Poznan, Poznan University of Medical Sciences, 60-355 Poznan, Poland; 2 Department of Clinical Pharmacy and Biopharmacy, Poznan University of Medical Sciences, 60-806 Poznan, Poland; 3 Department of Endocrinology, Metabolism and Internal Medicine, Poznan University of Medical Sciences, 60-355 Poznan, Poland; kktlacka@gmail.com

**Keywords:** hyperthyroidism, beta-blockers, thyroid cancer, pregnancy, amiodarone-induced hyperthyroidism

## Abstract

β-blockers have found wide application in cardiology, neurology, psychiatry, anaesthesiology, and endocrinology. Due to the reduction in excessive reactivity of the peripheral sympathetic nervous system, they have been used in the treatment of symptoms of hyperthyroidism. Significant efficacy in alleviating neuro-psychiatric symptoms associated with hyperthyroidism is attributed to propranolol, while cardiac symptoms are alleviated by both non-selective and cardioselective β-blockers. The aim of our study is to collect and summarise the existing knowledge on the role of β-blockers in patients with hyperthyroidism, with particular emphasis on pregnant patients, the group with iatrogenic hyperthyroidism, and patients after amiodarone. Due to their favourable safety profile, they appear to be a beneficial supplementary therapy to the treatment of hyperthyroidism in pregnant patients. β-blockers are also used in the treatment of complications of hyperthyroidism after amiodarone administration. They may influence the therapeutic process of amiodarone-induced hyperthyroidism itself, as well as being a therapeutic alternative to amiodarone in a cardiovascular context. By alleviating the symptoms associated with high doses of L-thyroxine, which are used, e.g., in. patients with thyroid cancer, β-blockers may make it possible to maintain low TSH values.

## 1. Introduction

β-blockers belong to a group of drugs that are widely used in cardiology, neurology, psychiatry, anaesthesiology, ophthalmology and endocrinology [1]. This group of drugs acts by antagonistically competing for the receptor binding site for endogenous catecholamines such as adrenaline and noradrenaline, which mediate the sympathetic nervous system response [2]. The binding of the β-blocker molecule to the receptor limits the action of catecholamines, reducing the effect of the sympathetic nervous system on the various tissues and organs under its influence [2]. 

Catecholamine receptors are found in various tissues, such as heart muscle, arterial walls, skeletal muscle, smooth muscle, bronchioles, kidneys, and adipose tissue [3]. β-blockers have the effect of reducing heart rate (HR), thereby increasing myocardial oxygen sensitivity and preventing ischaemic episodes [4]. Moreover, β-blockers can also reduce renin synthesis in the kidneys, thereby reducing pathological myocardial remodelling under the influence of aldosterone [5]. In addition, they have been shown to reduce the number of migraine episodes [6] or alleviate the symptoms of panic attacks [7]. Due to their action of reducing excessive reactivity of the peripheral sympathetic nervous system, they have been used in the treatment of symptoms of hyperthyroidism [8]. 

Hyperthyroidism is defined as a syndrome of symptoms caused by an excess of thyroid hormones, characterised by HR acceleration, increased peripheral resistance, decreased cholesterol levels, and increased metabolism, as well as tremor or neuropsychiatric symptoms [9,10]. Excess thyroid hormones are an important factor affecting the cardiovascular system. Untreated increased levels of thyroid hormones can cause atrial fibrillation (AF), embolic events, muscle weakness, and, in rare cases, cardiovascular collapse and even death [8,10].

In patients with symptomatic hyperthyroidism, the inclusion of β-blockers is recommended [8]. A special group of patients who may benefit from the use of β-blockers are those with cardiac symptoms—AF, elevated HR, or ischaemic heart disease due to the cardioprotective function of β-blockers. In the study by Tagami et al., the authors compared a group of patients taking methimazole alone and a group taking methimazole together with a β-blocker. After 4 weeks, patients taking β-blockers presented lower HR, less shortness of breath and fatigue, and improved ‘physical functioning’ [11]. β-blockers not only alleviate the symptoms of hyperthyroidism but also affect the conversion of L-3,5,3′,5′-tetraiodothyronine (T4) to 3,5,3′-triiodothyronine (T3). High doses of drugs such as propranolol or nadolol inhibit this process, thereby lowering the level of T3, which actively affects tissues [8].

The aim of our study is to compile and summarise existing knowledge on the use of β-blockers in the treatment of hyperthyroidism. The following study describes the use, benefits, and complications of β-blocker treatment, with particular emphasis on pregnant patients, the iatrogenic hyperthyroidism group, and post-amiodarone patients.

## 2. β-Blockers’ Characterisation

β-blockers, or β-adrenergic blockers, are a group of drugs commonly used in the treatment of cardiovascular diseases and cardiac arrhythmias. Some of them, primarily propranolol, are also used in the treatment of hyperthyroidism and certain anxiety conditions. β-blockers act through four main effects: they reduce the force of cardiac contraction through a negative inotropic mechanism, reduce the heart rate (negative chronotropic), slow AV conduction (negative dromotropic), and reduce the excitability of myocardial cells (negative bathmotropic) [12,13]. β-blockers are classified according to their selectivity for β1 and β2 receptors and the presence of additional pharmacological properties [14,15,16,17,18,19,20,21,22,23,24,25,26,27,28,29,30,31,32,33,34,35,36,37,38,39,40,41,42,43,44,45,46,47,48,49]. Table 1 presents the characteristics of β-blockers.

The main adverse effects include bradycardia, hypotension, fatigue, drowsiness, sleep disturbances, depression, bronchospasm (non-selective), impotence, and masking of hypoglycaemia symptoms [14,15,16,17,18,19,20,21,22,23,24,25,26,27,28,29,30,31,32,33]. Contraindications include shock, bradycardia, atrioventricular block, sick sinus syndrome, bronchial asthma, chronic obstructive pulmonary disease, severe heart failure in the course of decompensation, severe peripheral circulatory disorders, and allergies [34,35,36,37,38,39,40,41,42,43,44,45,46,47,48,49].

## 3. β-Blockers and Thyroid Hormone Metabolism

**Figure 1:**
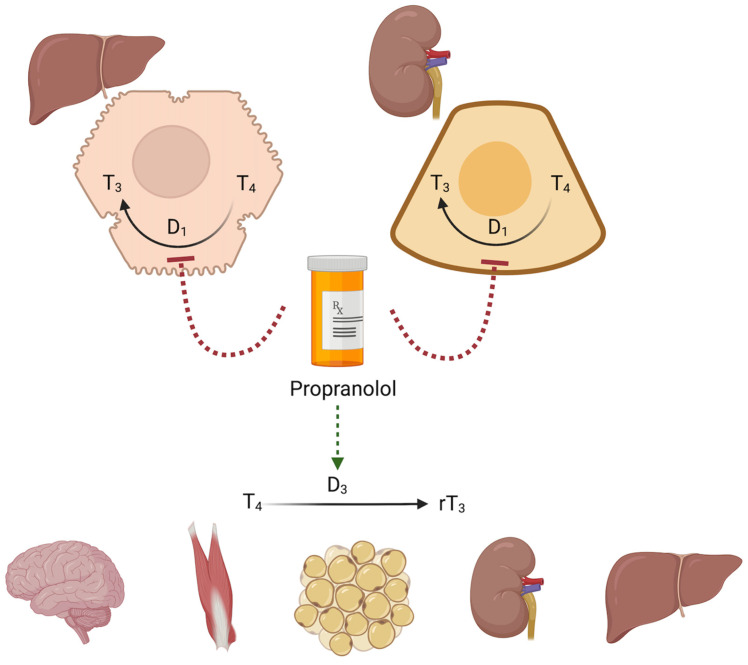
Propranolol and thyroid hormone metabolism [49,50,51,52]. T_3_—3,5,3′-triiodothyronine, T_4_—L-3,5,3′,5′-tetraiodothyronine, rT3—reverse T3 (inactive), D_1_—deiodinase type 1, and D_3_—deiodinase type 3. The green dashed arrow represents the activating effect; the red dashed lines represent the inhibitory effect. Created in BioRender. Miedziaszczyk, M. (2025) https://BioRender.com/3m58akt.

One of the most important mechanisms of action of β-blockers—particularly propranolol—is the inhibition of the conversion of L-3,5,3′,5′-tetraiodothyronine, T4, to active 3,5,3′-triiodothyronine, T3, in peripheral tissues, primarily the liver and kidneys [50,51]. This process is catalysed by deiodinase type 1 (D1). Propranolol reduces the activity of this enzyme, leading to a decrease in free T3 (FT3) levels and an increase in reverse T3 (rT3), an inactive metabolite [52]. Thyroid hormone metabolism is illustrated in Figure 1. Propranolol does not directly affect the production of T3 and T4 in the thyroid gland. They do not inhibit the enzymes responsible for tyrosine iodination or thyroglobulin synthesis, so drugs of this class cannot replace thyreostatic drugs [50]. A decrease in peripheral T3 levels results in a reduced effect on target organs. β-blockers are also effective in alleviating the symptoms of hyperthyroidism, regardless of T3/T4 levels, because they block the influence of thyroid hormones on the sympathetic nervous system and reduce symptoms of tachycardia, tremor, excessive sweating, anxiety, and fear [53]. It is worth noting that by blocking the conversion of T4 to T3, propranolol can lower FT3 levels in the blood without affecting TSH and free T4 (FT4) [50]. 

## 4. Hyperthyroidism 

Hyperthyroidism is defined as excessively high serum levels of thyroid hormones and excessive stimulation of thyroid hormone-sensitive tissues [54]. The prevalence of hyperthyroidism is estimated at 0.2–2.5% (in iodine-sufficient countries) [55]. Hyperthyroidism can be divided into overt or subclinical, depending on the biochemical presentation. Subclinical hyperthyroidism is manifested by low or undetectable serum TSH levels with normal T3 and free T4 levels. Overt hyperthyroidism is usually manifested by undetectable TSH with increased serum T3 and/or free T4 levels [54]. 

Various mechanisms can lead to excessive thyroid hormone levels. The most common cause of hyperthyroidism is Graves’ disease, in which excessive stimulation of the thyroid gland by a factor such as anti-TRAB antibodies leads to increased production and release of thyroid hormones [56]. Another cause may be the autonomous secretion of excess hormones by thyroid nodules. Toxic nodular disease is more common in regions with iodine deficiency and is the second most common cause of hyperthyroidism. Thyroid nodule cells acquire mutations in somatic genes responsible for regulating hormone synthesis, leading to autonomous hypersecretion of thyroid hormones [57]. Autoimmune, infectious, chemical, or mechanical factors can lead to the passive release of stored thyroid hormones in excessive amounts due to damage to the cells of the thyroid gland [8,58]. Painless thyroiditis with symptoms of hyperthyroidism can also occur during treatment with lithium [59] or tyrosine kinase inhibitors [60]. A particularly important drug whose use can trigger symptoms of hyperthyroidism is amiodarone. Amiodarone’s molecule contains iodine, whose excess may lead to hyperthyroidism, especially in patients with nodular or autoimmune thyroid disorders. Also, 5–10% of patients treated with amiodarone develop painless destructive thyroiditis [61]. Another cause of excess thyroid hormones may be endogenous production associated with ovarian cancer (struma ovarii) [62] or differentiated thyroid cancer [8]. Symptoms of hyperthyroidism can also be caused by too high a dose of L-thyroxine [63].

**Figure 2:**
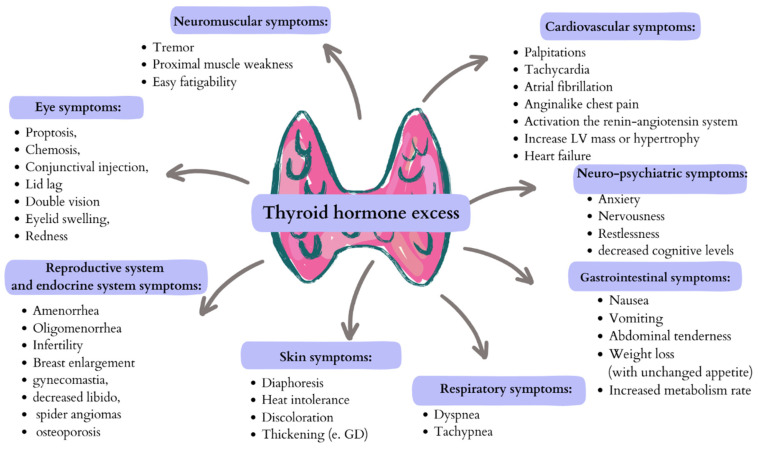
The possible clinical presentations of excess thyroid hormones. Created in Canva by W. Szybiak-Skora (2025) https://www.canva.com/design/DAGgUF7KVck/6jL3Cv9_-zGJJ783a2b3wQ/edit.

The aetiology of thyrotoxicosis can be assessed by measuring the ratio of total T3 to total T4. A hyperactivated thyroid gland synthesises more T3 than T4; T3 will be increased above the upper limit of normal more than T4. In the case of thyrotoxicosis caused by thyroiditis, T4 is elevated more than T3. In the study conducted by Carle et al., a group of patients with Graves’ disease and nodular goitre presented a ratio of total T3 to total T4 (ng/μg) >20 [64]. Shigemasa et al. established that in a group of patients with painless or postpartum thyroiditis, the ratio was <20. An increased T4-to-T3 ratio may be seen in thyrotoxicosis connected with exogenous L-thyroxine [63]. The clinical symptoms of excess thyroid hormones are shown in Figure 2.

## 5. Efficacy of β-Blockers in Controlling Symptoms Caused by Excess Thyroid Hormones

### 5.1. Psychiatric and Neurological Symptoms and β-Blockers 

Tremors, anxiety, and emotional lability are neuro-psychiatric consequences of elevated serum levels of thyroid hormones. Nervousness and increased sympathetic nervous system arousal are more often presented by groups of younger patients [65,66,67,68].

One of the drugs used to treat anxiety symptoms is propranolol. This non-cardioselective sympatholytic β-blocker crosses the blood–brain barrier. Moreover, propranolol is a weak indirect α1-adrenoceptor agonist [69,70], and it has been proven that it may act as a weak antagonist of certain serotonin receptors, 5-HT1A, 5-HT1B, and 5-HT2B, thus presenting an anxiolytic effect [71]. The efficacy of propranolol for anxiety was confirmed in a meta-analysis by Lonergan et al. In comparison with the placebo group, propranolol applied before memory consolidation reduced recall for negatively valenced stories and pictures [72].

Hyperthyroidism may also influence cognitive function. In the study conducted by Kudrjavcev et al., a group of elderly patients with hyperthyroidism presented dementia and confusion in 33% and 18% of patients, respectively [68]. Also, in the younger group of patients with hyperthyroidism, a reduced level of cognitive function was found compared to the control group of the same age [68]. Furthermore, studies conducted by Jaracz et al. provide evidence that neuropsychological disorders may also occur in patients with hyperthyroidism induced by exogenous thyroxine. Patients with differentiated thyroid cancer who were treated with TSH-suppressive therapy using L-thyroxine showed significantly lower scores on tests assessing executive function, psychomotor speed, and attention compared to the control group [73]. The β-blocker drug propranolol also appears to be useful in the treatment of cognitive impairment in patients with elevated thyroid hormone levels. In a study conducted by Faigel, the author demonstrated the efficacy of a single dose of propranolol given before the Scholastic Aptitude Test. In patients with anxiety-related cognitive impairment, the use of 40 mg propranolol improved test results compared with tests conducted without the drug. [74].

Excess thyroid hormone can lead to tremor [65], which is defined as involuntary, rhythmic, and oscillating movement of a body segment [75,76]. The tremor symptoms can be partially relieved with symptomatic treatment. Propranolol monotherapy (at doses of 120–240 mg/day) has been shown to effectively reduce the severity of tremor associated with essential tremor [77]. Randomised clinical trials have shown that propranolol can reduce tremor amplitude by up to approximately 50% [78]. Furthermore, the combination of propranolol with primidone reduces tremor amplitude by 60%, thus providing additional benefits [79]. 

**Figure 3:**
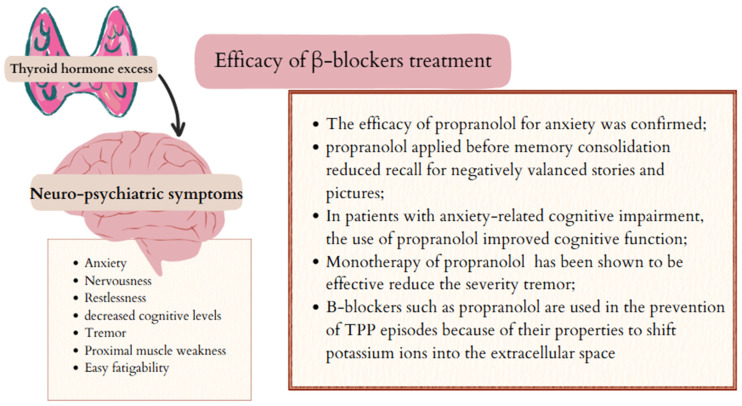
Efficacy of β-blockers in neuropsychiatric disorders induced by excess thyroid hormones. TPP—thyrotoxic periodic paralysis. Created in Canva by W. Szybiak-Skora (2025) https://www.canva.com/design/DAGgUF7KVck/6jL3Cv9_-zGJJ783a2b3wQ/edit.

Patients suffering from hyperthyroidism often present with pain and weakness in the proximal muscles of the shoulder and pelvic girdle. They report weakness, difficulty combing their hair, or climbing stairs [65]. In addition, there are reports in the literature of thyrotoxic periodic paralysis (TPP) is a rare complication of hyperthyroidism characterised by episodes of muscle weakness and hypokalemia [80]. The severity of attacks varies from mild weakness to quadriplegia or total paralysis. It has been proven that patients suffering from TPP have an increased amount and activity of the Na-K-ATPase pump compared to healthy controls [81]. Thyroid hormone, insulin, and the beta-adrenergic catecholamine can lead to increased muscle pump activity in skeletal muscle. This process causes potassium to shift into cells, leading to low serum potassium levels. Muscle weakness resolves as potassium returns to the extracellular space [82]. Treatment of TPP involves treating hyperthyroidism with standard methods such as antithyroid drugs, β-blockers, surgery, or iodine therapy. The patient described in the study by Barahon et al. was free of TPP symptoms after one year of treatment with metamizole and propranolol. β-blockers such as propranolol are used in the prevention of TPP episodes because of their ability to shift potassium ions into the extracellular space [80]. The effectiveness of β-blockers in neuropsychiatric disorders caused by excess thyroid hormones is presented in Figure 3.

### 5.2. Cardiovascular Symptoms and β-Blockers 

Thyroid hormones affect the cardiovascular system. The most common cardiovascular symptoms of hyperthyroidism include palpitations and an increased heart rate, not only during exercise but also at rest [65,83]. Sinus tachycardia is most frequently diagnosed on electrocardiography in patients with hyperthyroidism. In older patients with coronary artery disease or additional valvular dysfunction, AF is more commonly diagnosed [84]. Arrhythmias such as ventricular tachycardia, supraventricular tachycardia, or atrial flutter are diagnosed less frequently [85].

Increased serum concentrations of thyroid hormones increase the expression of myocardial sarcoplasmic reticulum calcium-dependent adenosine triphosphatase (ATP) [8,65]. Increased ATP concentration in the heart leads to increased chronotropy (increased HR) and inotropy (contractility) of the heart, which in turn causes increased left ventricular ejection fraction (EF) and cardiac output (CO) [8,65].

The action of thyroid hormones on the tissues induces a state of hypermetabolism, which is associated with increased metabolic process, increased oxygen consumption, and thus an increase in metabolic products [65]. An inadequate supply of oxygen in relation to demand and a concomitant state of myocardial overactivity can lead to the onset of ischaemic pain and deterioration of exercise tolerance [86].

Excess products of intensive metabolism, such as lactic acid, for example, lead to vasodilation and a decrease in peripheral resistance. Renal blood flow is then reduced, and the renin–angiotensin–aldosterone system (RAAS) is activated [87]. There is water and sodium retention in the body and increased levels of aldosterone and angiotensin II, which show adverse effects on myocardial remodelling. As a result of the increased activity of the RAA system, there is an increase in circulating blood volume and preload for the heart [86]. Excess thyroid hormones also cause an increase in erythrocyte mass, and changes in T4 levels have been correlated with changes in erythropoietin levels [88]. Volume overload and the direct activity of thyroid hormones on the increase in cellular protein synthesis, especially in cardiac muscle cells, lead to cardiac hypertrophy [89].

Long-term haemodynamic imbalances associated with excess thyroid hormones lead to myocardial remodelling, which may eventually lead to symptoms of congestive heart failure [65]. Myocardial hypertrophy leads to impaired compliance and excessive inflexibility of the left ventricle, which reduces ventricular filling, especially when supraventricular tachyarrhythmia occurs [90]. Left ventricular diastolic dysfunction occurs in approximately 30% of patients with clinically overt hyperthyroidism and increases with age [90]. A study by Nanchen et al. showed that patients aged 70–82 years with subclinical hyperthyroidism (reduced TSH and normal FT4) have an increased risk of developing heart failure (HF) [91]. In addition, there is an elevation in total circulating blood volume and plasma volume as a result of thyroid hormone activity. This contributes to raised left ventricular filling pressures, impaired systolic function, and reduced minute volume [86]. 

The incidence of heart failure in patients with hyperthyroidism usually correlates with the presence of pre-existing heart disease, such as arrhythmia, hypertension, or ischaemic heart disease [65], but HF can also manifest in patients with hyperthyroidism but without structural heart disease. In this case, HF progresses with high cardiac output [92]. This type of failure is more likely to occur in patients in the initial phase of hyperthyroidism, when abnormalities such as tachycardia, decreased peripheral resistance, increased stroke volume, and consequent systolic hypertension with decreased diastolic pressure predominate [93]. Haemodynamic abnormalities in HF with high cardiac output include increased minute volume and increased myocardial contractility. This type of HF depends largely on sinus tachycardia or AF with rapid ventricular activity and is referred to as tachyarrhythmic cardiomyopathy [92]. In this case, the patient may present with various symptoms of HF: easy fatigue, features of peripheral stasis, a feeling of breathlessness at rest, pleural fluid, and increased pulmonary artery pressure [92].

Recommendations for the treatment of hyperthyroidism include the inclusion of β-blockers in the treatment of patients with hyperthyroidism. β-blocker drugs (e.g., propranolol or nadolol), by blocking the conversion of T4 to T3, reduce the production of the more tissue-active T3, thereby participating in the direct treatment of the cause of hyperthyroidism and contributing to the achievement of euthyroidism [8].

A particular group of patients who may benefit from treatment with β-blockers are those with cardiovascular symptoms, due to the broad effects of this group of drugs on the myocardium [8]. β-blocker drugs prevent sympathetic-dependent dysrhythmias and slow conduction in the sinus and atrioventricular nodes. The reduced nodal conduction has the effect of reducing myocardial chronotropy and thus reducing HR [94]. A study by Xie et al. demonstrated that the addition of propranolol to the treatment of hyperthyroidism can reduce heart rate without additional adverse effects. Propranolol addition gave significantly better HR-control compared to only metamizole treatment [4]. 

Reduced HR, decreased myocardial hyperkinesis, and prolonged nodal conduction time result in prolonged diastole, improved cardiomyocyte blood supply, and reduced myocardial oxygen demand. The use of β-blockers reduces the incidence of ischaemic symptoms and also reduces the risk of acute myocardial ischaemia [95]. Reducing adrenergic stimulation prevents the development of myocardial hypertrophy and pathological remodelling. A study conducted by Blumenfeld et al. quantified the effect of β-blockers on the RAAS and examined their effect on the ratio of plasma renin activity to total renin. The results indicate that β-blockers reduce serum angiotensin II levels, in parallel with a significant reduction in plasma renin activity and urinary aldosterone levels in normotensive and hypertensive patients. The reduction in RAAS activity is associated with a reduction in the effects of angiotensin II and aldosterone on the myocardium, thereby reducing their adverse effects on myocardial remodelling [96]. In a study by Biondi et al. on suppressive thyroxine treatment, the addition of a cardiac β1-adrenergic receptor blocker to suppressive treatment was shown to significantly reduce left ventricular hypertrophy [97]. 

**Figure 4:**
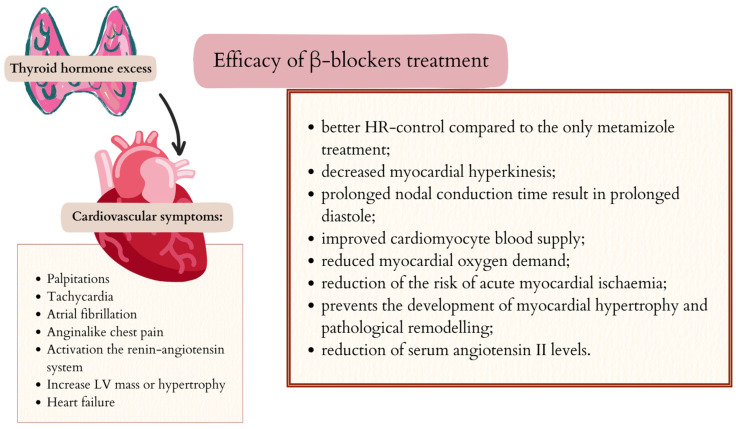
Efficacy of β-blockers in cardiac disorders induced by excess thyroid hormones. Created in Canva by W. Szybiak-Skora (2025) https://www.canva.com/design/DAGgUF7KVck/6jL3Cv9_-zGJJ783a2b3wQ/edit.

Cardioselective β-blockers (metoprolol, atenolol, and esmolol) have been shown to be as effective as non-cardioselective ones in controlling exaggerated cardiovascular responses in thyrotoxicosis [98]. A study by Palmieri et al. observed the effect of acute β1 adrenergic blockade (bisoprolol) on myocardial contractility and total arterial stiffness in patients with thyrotoxicosis. It has been shown that there is a sustained increase in preload in hyperthyroidism. Patients treated with bisoprolol showed a reduction in cardiovascular hyperkinesia, as manifested by a decrease in heart rate. Specific blockade of β1 adrenergic receptors with bisoprolol was found to lead to normalisation of total arterial stiffness [99]. The effectiveness of β-blockers in the treatment of heart disorders caused by excess thyroid hormones is shown in Figure 4.

### 5.3. Respiratory Symptoms and β-Blockers

Patients with excess thyroid hormones experience weakened respiratory muscles, decreased lung compliance, and increased airway resistance [65]. The state of hyperthyroidism in patients suffering from asthma may exacerbate the symptoms of this disease [100]. Furthermore, a study by Wang et al. proved that hyperthyroidism was associated with an increased risk of asthma development [101]. It is important to be cautious about pulmonary symptoms in patients with hyperthyroidism who are treated with β-blockers, due to their broncho-constrictive effects. Patients with co-occurring asthma and hyperthyroidism may benefit from β1-selective β-blocker therapy.

Changes in the respiratory system under the influence of thyroid hormones can exacerbate the feeling of dyspnoea that occurs in patients with hyperthyroidism and cardiovascular alterations. HF with high cardiac output may lead to pulmonary artery dilatation and the development of pulmonary hypertension [102]. The role of β-blockers in the treatment of PAH remains unclear and requires more research. The sympathetic nervous system and adrenergic stimulation maintain the disturbed haemodynamic balance in patients with PAH. However, current studies show that β-blockers may have a beneficial effect on the right side of the heart and pulmonary artery morphology [103]. In animal models in which PAH was induced in vivo, β-blockers improved diastolic function, rebalanced metabolic enzymes in myocardial cells, and improved contractility in PAH. These results indicate that β-blockers may have a beneficial effect on the course of PAH by promoting right ventricular function [104,105]; however, more studies are still needed.

## 6. Treatment with β-Blockers in Specific Clinical Situations Associated with Excess Thyroid Hormones

### 6.1. Levothyroxine Suppressive Therapy in Differentiated Thyroid Cancer

Thyroid cancer is diagnosed in approximately 44,000 people per year in the US population, with a 5-year survival rate of approximately 98.5% [106,107]. The most common thyroid cancer is papillary thyroid cancer, which accounts for approximately 84% of all thyroid cancer types [106]. Both papillary carcinoma and forms of follicular carcinoma (≈4%) and oncocytic carcinoma (≈2%) are classified as highly differentiated carcinomas that originate from follicular thyroid cells. Differentiated thyroid cancer can develop in a healthy thyroid, in a gland affected by Hashimoto’s thyroiditis, in a multinodular goitre, in patients with a single nodule, or in patients with Graves’ disease (rarely) [108,109]. Poorly differentiated thyroid carcinoma (≈5%) and anaplastic thyroid carcinoma (≈1%) are aggressive forms of thyroid carcinoma originating from follicular thyroid cells [107]. In addition to tumours originating from follicular cells, parafollicular C-cell tumours can also develop in the thyroid gland. Such tumours include medullary thyroid cancer (≈4%), whose cells, like C cells, secrete a significant level of calcitonin [107].

Thyroid carcinomas do not usually present with specific clinical symptoms. They may cause an enlargement of the neck circumference or present as a neck region tumour, perceptible on the patient’s self-examination or on the doctor’s palpation examination. However, thyroid carcinomas are mostly detected on incidental imaging [110]. The primary form of treatment for thyroid cancer is thyroidectomy. For small lesions <1 cm, an observation–control strategy can be used [111]. In patients suffering from cancerous lesions originating from iodochondria cells, in which surgery alone was insufficient, complementary radioiodine therapy is used [112]. Patients with advanced thyroid cancer and a specific cancer-causing genetic mutation (BRAF, RET, NTRK, or MEK) may benefit therapeutically from targeted therapies such as dabrafenib and selpercatinib [107]. 

Patients whose thyroid cancer from follicular thyroid cells has been treated by surgical resection are usually treated with L-thyroxine not only to supplement the postoperative deficiency but also to decrease TSH levels [107]. Patients with a diagnosis of poorly differentiated and anaplastic thyroid cancer or medullary thyroid carcinoma require only substitutive L-thyroxin doses [113].

Tumour cells derived from follicular thyroid cells have receptors for TSH. Excessive TSH levels, induced by reduced levels of thyroid hormones after surgery, can stimulate residual tumour cells to continue to grow and divide, which may constitute a risk for the development of tumour recurrence. The degree of TSH suppression depends on the risk of cancer recurrence [107,114]. The individual targets for TSH suppression are shown in Table 2.

Nevertheless, the use of suppressive doses of L-thyroxine in this group of patients is associated with the risk of developing symptoms of iatrogenic hyperthyroidism [114]. Therefore, it is important that the balance between oncological TSH suppression and the risk of adverse effects of L-thyroxine is maintained. A particular group of patients at increased risk of adverse reactions to L-thyroxine therapy is elderly patients [116]. They may develop cardiovascular complications or bone disorders associated with osteoporotic changes [114]. Diagnosis among patients over 60 years of age is made more difficult due to poorer symptom perception and less characteristic clinical presentation of cardiac symptoms [116]. In older patients, the main manifestation of hyperthyroidism associated with TSH suppression is AF. Prospective studies have confirmed an increased risk of developing AF in patients on TSH-suppressive therapy compared to controls [117,118]. A meta-analysis conducted by Haentjens et al. showed that untreated subclinical hyperthyroidism is more destructive in patients with comorbidities (heart disease or diabetes mellitus) [119]. Subclinical hyperthyroidism individuals present a 41% increase in relative mortality from all causes in comparison to euthyroid controls [119]. Furthermore, the increased mortality in patients with hyperthyroidism increases after 60 years of age [119].

Young and middle-aged patients receiving long-term therapy with suppressive doses of l-thyroxine may experience cardiovascular alterations such as increased heart rate, increased left ventricular mass, increased mean arterial pressure, and diastolic dysfunction [120]. Detection and control of these changes in this group of patients is important because of the increased risk of developing subsequent disease and death from cardiovascular causes [107].

Patients receiving TSH-suppressive treatment with l-thyroxine who present with cardiovascular symptoms or are at cardiovascular risk may benefit from the inclusion of β-blocker treatment. A study conducted by Biondi et al. established that the inclusion of bisoprolol in L-thyroxine suppressive therapy resulted in the normalisation of HR, the normalisation of atrial arrhythmias, and a reduction in palpitation feeling [97]. The echocardiographic data showed that after 6 months of suppression TSH therapy with added bisoprolol, left ventricular mass index normalised, and the indices of left ventricular systolic function were reduced, with a tendency to normalise [97]. 

In the other investigation conducted by Gullu et al., it was found that isovolumetric relaxation time decreased in a group of patients with TSH suppression after inclusion of atenolol [121]. 

Similarly, in the study conducted by Fazio et al., the authors concluded that long-term suppressive TSH therapy with L-thyroxine led to increased myocardial mass and cardiac hypertrophy, which resulted in relevant diastolic dysfunction [122]. Both myocardial hypertrophy and diastolic dysfunction are significantly improved by adrenergic β-blockade [122]. The results of the aforementioned investigations are summarised in Table 3.

### 6.2. Pregnancy

Clinically overt hyperthyroidism complicates between 0.1% and 0.4% of pregnancies. The most common causes are gestational thyrotoxicosis or Graves–Basedow disease [123]. 

The corpus luteum and placenta during pregnancy produce human chorionic gonadotropin (hCG), the serum level of which peaks around the 10th week of gestation. The B subunit of hCG, with a structure comparable to TSH, can stimulate the receptor for TSH, thereby causing an increase in the production and secretion of thyroid hormones and a decrease in serum TSH levels [124]. A study by Glinoer et al. examined 300 pregnant women, 18% of whom presented reduced TSH levels during the first trimester. Of these, 50% had TSH levels <0.05 mU/L, indicating complete TSH suppression. In this group of patients, elevated FT4 values appeared in 10% [125].

Graves’ disease, first appearing during pregnancy, can most often develop during the first months of pregnancy (first trimester). The most significant risk of developing the disease postpartum is during the seventh to ninth month after delivery [124]. Patients diagnosed with Graves–Basedow disease before pregnancy have the greatest risk of exacerbation of the disease by the 15th week of pregnancy [126]. Conversely, patients in the last trimester of pregnancy have the lowest risk of exacerbating existing Graves’ disease or developing the disease due to the immune tolerance that develops during this time [126].

The treatment of hyperthyroidism in pregnancy depends on its aetiology and the individual characteristics of the patients. In patients with gestational thyrotoxicosis, antithyroid drugs (ATDs) are not recommended due to the transient nature of the abnormality and the possible adverse effects of these drugs. A form of treatment that could be considered in this group of patients is the inclusion of β-blockers to control the symptoms of transient hyperthyroidism [124]. The treatment of pregnant patients with Graves’ disease presents the clinical challenge of maintaining control of normal thyroid hormone levels not only in the mother but also in the foetus. It is important to remember that both anti-TSH antibodies and ATDs cross the placenta [124]. Therefore, it is important to minimise the adverse effects of the drugs on the developing foetus and the course of pregnancy. Antithyroid drugs are the mainstay of therapy for Graves’ disease in pregnancy [127]. However, both Propylthiouracil and methimazole show teratogenic effects.

Foetal exposure to methimazole (antithyroid medication), especially between 6 and 10 weeks of gestation, can cause severe embryopathy in 2 to 4% of foetuses [124]. Malformations may include skin aplasia, dysmorphic facial features, ventricular septal defects, oesophageal atresia, biliary atresia, umbilical atresia, and omphalomesenteric duct anomalies [128,129]. Propylthiouracil has been found to be safe in pregnancy and is the preferred drug during the first trimester of pregnancy, during which organogenesis occurs. However, in a population-based study conducted by Andersen et al., excessive birth defects were identified in 2% to 3% of children exposed to PTU, mainly facial and neck malformations [129]. 

In view of the numerous adverse effects of ATDs, it is worth considering the role of β-blocker therapy in pregnant women. β-Blockers can cross the placenta and affect the developing foetus, but they also have an effect on the uterus, influencing the progression of labour. Some studies have confirmed the occurrence of mild and transient hypoglycaemia, bradycardia, hypotension, and apnoea in newborns after the use of propranolol in the last weeks of pregnancy [130,131,132,133]. Studies also suggest that the use of β-blockers, especially atenolol, causes prolonged contraction of the uterine myometrium, which reduces flow through the vessels of the placenta, thereby limiting the growth of the developing foetus [8,134]. A study by Kubota et al. established a statistically significant increased risk of small for gestational age (SGA) in a group of pregnant women treated with β-blockers in comparison to controls [135]. A study by Sherif et al. showed that a higher rate of spontaneous abortion was observed in the group of patients treated with propranolol and carbimazole compared to the group treated with carbimazole alone. However, due to the limited number of patients and the diagnostic limitations of the study, the effect of β-blockers on the risk of spontaneous abortions is not clearly defined [136]. Research has shown, however, that the use of β-blockers in pregnancy may be safe, especially when used short term and for specific indications [137,138,139]. In a meta-analysis conducted by Wu et al., the authors analysed 20 observational studies and established that exposure to β-blockers during early pregnancy does not appear to be associated with congenital malformations or heart malformations in offspring [140]. Selective β-blockers also appear to show a greater safety profile for pregnant patients [137,138,139]. 

According to current guidelines, treatment with propranolol 10–20 mg every 8 h or metoprolol 100 mg once daily for short-term use to relieve symptoms in pregnant women with thyrotoxicosis may be considered safe [8]. Atenolol should be avoided [8,141]. When considering the use of β-blockers during pregnancy, the changing pharmacokinetics (PK) of drugs in the first, second, and third trimesters should also be taken into account, especially since some drugs in this group are lipophilic (e.g., propranolol and timolol) and some are hydrophilic (e.g., atenolol and sotalol) [75], which may determine how they are dosed. For example, the dosage of labetalol should be adjusted to the patient’s weight during pregnancy [142]. The physicochemical property of β-blockers affects the degree of penetration through the placenta and ultimately the safety of the therapy. Changes in the PK of β-blockers that should be taken into account include enhanced first-pass metabolism, increase in body fluid volume, cardiac output, decrease in albumin concentration, induction of liver enzymes (CYP3A4, CYP2D6, CYP2C9, UGT1A4, and UGT2B7), inhibition of CYP1A2 and CYP2C19, and increased glomerular filtration [142,143]. It is possible that, due to the aforementioned changes in enzyme and drug transporter activity during pregnancy, β-blocker doses should be increased to avoid subtherapeutic concentrations, but this requires further pharmacokinetic and pharmacodynamic studies. The effects of β-blockers on pregnancy and breastfeeding are presented in Table 4.

### 6.3. Amiodarone-Induced Thyroid Disease 

Amiodarone is a medication used in cardiology to treat atrial and ventricular arrhythmias. It is often used in patients in whom other therapeutic modalities have been ineffective [61]. Unlike other antiarrhythmic medications, it can be used in patients who present with ischaemic heart disease or structural heart disease such as myocardial hypertrophy [61].

Amiodarone, according to the Vaughan–Williams classification, is a class III antiarrhythmic drug. Its action is mainly through the inhibition of K+/Na+ ATPase, which leads to a delay in phase 3 depolarisation and a prolongation of the refraction period [61,144]. In addition, its activity can block Na channels and decrease conduction, reduce the number of β-adrenergic receptors, and affect Ca-mediated processes [61]. The amiodarone molecule resembles the levothyroxine molecule in its structure. Similarly to thyroid hormones, it contains an iodine atom [145]. The use of a standard dose of 100–600 mg of amiodarone per day provides approximately 3–21 mg of iodine per day, which significantly exceeds the recommended daily dose of iodine [146]. Due to its structure and significant iodine content, the use of amiodarone therapy could cause adverse effects in the form of thyroid dysfunction. According to previous studies, approximately 10–20% of patients treated with amiodarone on short-term therapy may develop amiodarone-induced hyperthyroidism [147]. The risk of developing hyperthyroidism shows a particular increase in patients with iodine deficiency and among women [61,148]. Furthermore, this risk decreases in patients taking amiodarone chronically (min. 1 year), which may be due to adaptive mechanisms of the thyroid gland [147,148].

Amiodarone-induced hyperthyroidism (AIH) can be divided into two types: Type 1 AIH results from the Jod–Basedow effect and is diagnosed in patients with pre-existing thyroid disease [65]. Type 2 AIH is associated with damage to the thyroid gland by the presence of an inflammatory process involving pro-inflammatory cytokines in response to the amiodarone used [65]. In the treatment of AIH, antithyroid medications and iodine therapy are often ineffective, and the only method to reduce symptoms is to discontinue amiodarone [149]. Amiodarone discontinuation does not always result in the expected effects. Amiodarone has a β-adrenergic receptor blocking effect, so despite inducing AIT, it can also alleviate its symptoms. Due to its β-blocking activity, amiodarone withdrawal may paradoxically exacerbate AIT symptoms [150]. Furthermore, amiodarone has a long half-life, so its withdrawal will not have an immediate effect [151,152].

In light of the above data, the question arises as to whether patients being treated for AIH may benefit from the inclusion of β-blockers. Patients with AIT symptoms, according to guidelines, may benefit from the inclusion of β-blockers due to their ability to reduce hyperthyroid symptoms [153]. The use of β-blockers, especially cardioselective ones, instead of amiodarone as an arrhythmia treatment should be considered in the context of cardiovascular risk. 

Some patients may benefit from the use of dronedarone, which lacks the iodine molecule, instead of amiodarone. However, this medication is contraindicated in patients with New York Heart Association (NYHA) class IV heart failure or NYHA class II-III heart failure with recent decompensation [154]. In addition, in PA patients with arrhythmias that require treatment with amiodarone, ablation should be considered.

## 7. Conclusions

β-blockers are antiarrhythmic medications that are also used for non-cardiac indications. They are particularly used in the treatment of hyperthyroidism. 

Due to their peripheral effect of reducing sympathetic nervous system hyperactivity, they alleviate the symptoms of hyperthyroidism, such as palpitations, shortness of breath, symptoms of ischaemia, and cardiac hypertrophy. β-blockers also play an important role in controlling sinus rhythm in patients with atrial fibrillation, which is the most common arrhythmia in patients with hyperthyroidism. In addition, studies have also shown that propranolol can alleviate neurological and psychiatric symptoms associated with excess thyroid hormones, such as anxiety, restlessness, tremors, and concentration and attention disorders.

Due to their favourable safety profile, they appear to be a beneficial supplement to the treatment of hyperthyroidism in pregnant patients. β-blockers are also used in the treatment of complications of hyperthyroidism after amiodarone administration. They may influence the therapeutic process of amiodarone-induced hyperthyroidism itself, as well as being a therapeutic alternative to amiodarone in a cardiovascular context. By alleviating the symptoms associated with high doses of L-thyroxine, which are used, e.g., in patients with thyroid cancer, β-blockers may make it possible to maintain low TSH values.

## Figures and Tables

**Table 1:** β-blockers’ characterisation.

β-Blocker	Receptors	Lipophilicity	T4→T3 Inhibitor	Additional Effect
Propranolol	β1, β2	High	Yes	
Sotalol	β1, β2	Low	No	K+ channel blocker
Acebutolol	β1	Medium	No	ISA
Atenolol	β1	Low	No	Hydrophilic
Betaxolol	β1	Medium	No	
Bisoprolol	β1	Medium	No	
Celiprolol	β1	Medium	No	ISA
Esmolol	β1	Low	No	Short period of action
Metoprolol	β1	Medium	No	
Nebivolol	β1	High	No	NO action
Carvedilol	β1, α1	High	No	Antioxidant

**Table 2:** Individual targets for TSH suppression depend on the risk of recurrence [107,115].

Low-Risk <5% Risk of Recurrence	Intermediate-Risk 10–20% Risk of Recurrence	High-Risk 30–55% Risk of Recurrence
0.1–0.5 mlU/L if thyroglobin is detectable 0.5–2.0 mlU/L if thyroglobin is undetectable or lobectomy is performed	0.1–0.5 mlU/L only in high-risk patients or those who do not demonstrate excellent treatment response	<0.1 mlU/L only in high-risk patients or in patients who do not demonstrate excellent treatment response; in patients with persistent, clinically apparent DTC symptoms; with incomplete biochemical response according to the ATA—patients with no structural disease, but elevated stimulated Tg levels (>10 ng/mL) and/or elevated Tg levels on thyroxine suppression (>1 ng/mL), or an increased level of anti-Tg antibodies; those who are at high risk of recurrence and have no contraindications to suppressive therapy, or the benefits of therapy outweigh the risks of suppressive therapy; for those who are receiving complete suppressive therapy, the addition of a β-antagonist or angiotensin-converting enzyme inhibitor should be considered to prevent myocardial hypertrophy.

In patients with risk factors for hypothyroidism, such as high TSH before surgery, the presence of anti-thyroid antibodies (especially anti-TPO), and features of chronic thyroiditis on histopathological examination, appropriately higher doses of l-thyroxine should be used. DCT: differentiated thyroid cancer; ATA: American Thyroid Association.

**Table 3:** Influence of β-blockers on heart parameters in patients with thyroid cancer treated with suppressive doses of L-thyroxine.

Author, Year	β-Blockers with Dose	Heart Parameter	Results	*p*
Gullu, 2005 [121]	Atenolol 50 mg/day	Isovolumetric relaxation time (IVR)	IVR decreased (92 +/− 10 vs. 101 +/− 9 ms)	<0.05
		Left ventricular mass index (LVMI)	LVMI decreased (96 +/− 17 vs. 88 +/− 16 g/m^2^)	NS ^a^
		Diastolic diameters, early (VE) and late (VA)	VE and VA improved VE (0.72 +/− 0.12 vs. 0.79 +/− 0.2 m/s) VA (0.72 +/− 0.23 vs. 0.69 +/− 0.12 m/s)	NS
Biondi, 1996 [97]	Bisoprolol (4.25 +/− 0.4 mg/day)	Left ventricular ejection fraction (LVEF)	LVEF improved (63 +/− 2% to 53 +/− 2%)	<0.01
Fazio, 1995 [122]	Bisoprolol (4.25 +/− 1.2 mg/day)	Left ventricular mass index (LVMI)	LVMI decreased (80 +/− 18 vs. 95 +/− 19)	<0.001

^a^ NS—Not significant.

**Table 4:** Effect of β-blockers during pregnancy and breastfeeding.

β-Blocker	FDA Category	Breastfeeding Recommendation
Propranolol	C	No Human Data—Potential Toxicity
Sotalol	B	No Human Data—Potential Toxicity
Acebutolol	B/D II–III trimester	No Human Data—Potential Toxicity
Atenolol	D	No Human Data—Potential Toxicity
Betaxolol	C	No Human Data—Potential Toxicity
Bisoprolol	C	No Human Data—Potential Toxicity
Celiprolol	NA	N/A
Esmolol	C	No Human Data—Probably Compatible
Metoprolol	C	No Human Data—Potential Toxicity
Nebivolol	C	No Human Data—Potential Toxicity
Carvedilol	C	No Human Data—Probably Compatible

NA: Not available. B category—No risk in other studies: Animal reproduction studies have failed to demonstrate a risk to the foetus, and there are no adequate and well-controlled studies in pregnant women OR animal studies have shown an adverse effect, but adequate and well-controlled studies in pregnant women have failed to demonstrate a risk to the foetus in any trimester. C category—Risk not ruled out: Animal reproduction studies have shown an adverse effect on the foetus, and there are no adequate and well-controlled studies in humans, but potential benefits may warrant the use of the drug in pregnant women despite potential risks. D category—There is positive evidence of human foetal risk based on adverse reaction data from investigational or marketing experience or studies of humans, but the potential benefits from the use of the drug in pregnant women might be acceptable despite its potential risks.

## Data Availability

No new data were created or analysed in this study.
